# A novel polysaccharide in *Polygonatum kingianum*: structure elucidation, the activities of anti-inflammatory and the regulation of gut microbiota in vitro

**DOI:** 10.1007/s13659-025-00542-7

**Published:** 2025-09-02

**Authors:** Xiao Han, Xin-Xiu Ren, Dan-Yang Zhang, Qin-Feng Guo, Shi-Meng Li, Zhi-Long Xiu, Yue-Sheng Dong

**Affiliations:** 1 MOE Key Laboratory of Bio-Intelligent Manufacturing, School of Bioengineering, Dalian University of Technology, Dalian, 116024 Liaoning China; 2 College of Basic Medical Sciences, Dalian Medical University, Dalian, 116044 China

**Keywords:** *Polygonatum kingianum* Coll. et Hemsl., Polysaccharide, Structure elucidation, Bioactivity

## Abstract

Polysaccharides are the primary active constituents of *Polygonatum kingianum* Coll. et Hemsl. However, the comprehensive characterization of *P*. *kingianum* polysaccharides (PKP) remains scarce, impeding investigations into the structure–activity relationship. In this study, a novel polysaccharide, PKP1, was purified using Cellulose DE-52 and Sephadex G-50 column chromatography, and its complete structure was elucidated through monosaccharide composition analysis, methylation analysis, as well as 1D and 2D NMR analysis. The results revealed that PKP1 primarily comprised Fru and Glc, exhibiting a molecular weight of 5.3 × 10^3^ Da and a polymer dispersity index of 1.20. The completed structure of PKP1 consisted of β-D-Fru*f*-(2 → , → 1,2)-β-D-Fru*f*-(6 → , → 1)-β-D-Fru*f*-(2 → and → 1)-α-D-Glc*p*-(6 → as the main chain sugar residues, with β-D-Fru*f*-(2 → and → 2)-β-D-Fru*f*-(6 → serving as the side chains sugar residues. The detailed structure of PKP1 suggested it is a novel Fru-dominated neutral polysaccharide. Biological assays indicated that PKP1 significantly reduced the levels of NO, IL-6, and TNF-α in RAW264.7 macrophages, while also exerting regulatory effects on the gut microbiota structure and its metabolites in vitro. Our findings enriched the understanding of the structural characteristics of *P*. *kingianum* polysaccharides and laid a solid foundation for considering *P*. *kingianum* as a potential functional food supplement.

**Graphical Abstract:**

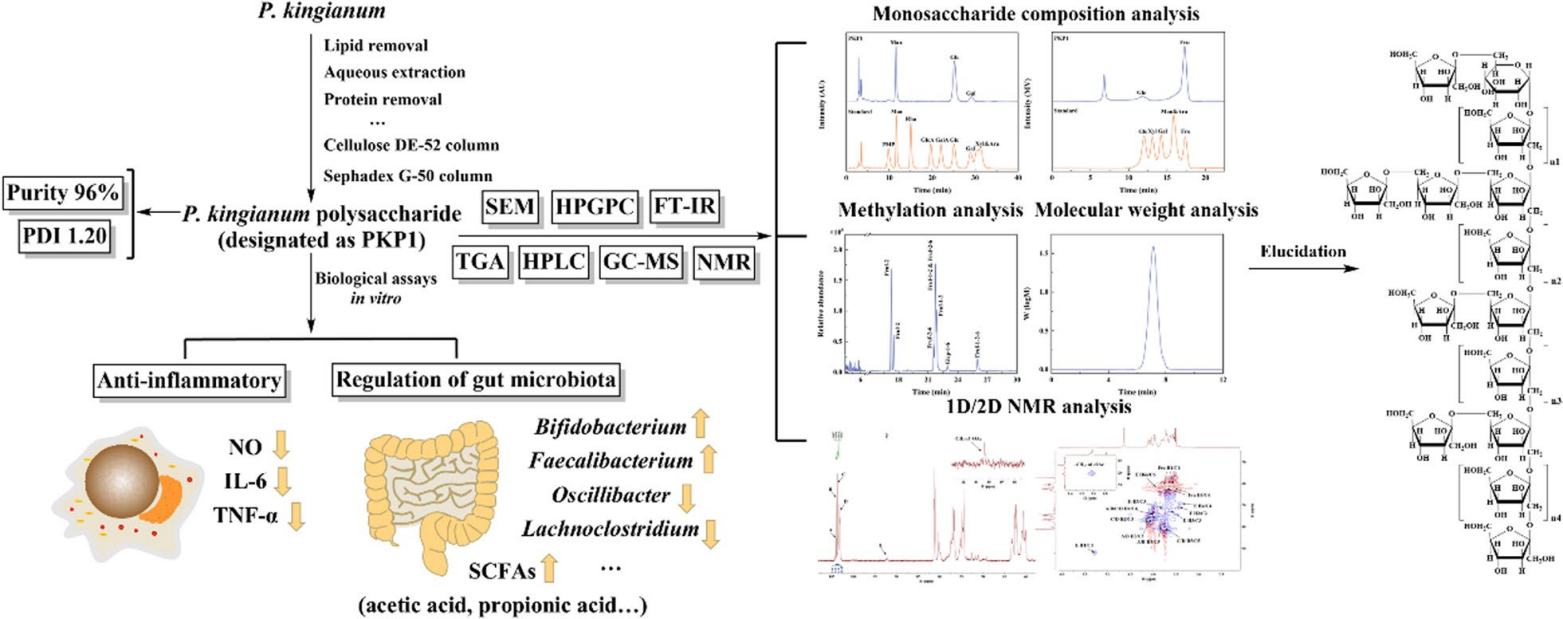

**Supplementary Information:**

The online version contains supplementary material available at 10.1007/s13659-025-00542-7.

## Introduction

Polysaccharides are found in almost all living organisms and represent a kind of naturally occurring active compound [1, 2]. With the deepening of research into polysaccharides, it has been demonstrated that the bioactivity of polysaccharides is closely related to their chemical structure [3, 4]. Therefore, elucidating the structure of polysaccharides is a prerequisite for understanding structure–activity relationships.

*Polygonatum kingianum* Coll. et Hemsl. belongs to the genus Polygonatum in the family Asparagaceae, primarily distributed in Yunnan, Sichuan, and Guizhou provinces of China [5]. Previously misidentified as *Polygonatum sibiricum* Red. or *Polygonatum cyrtonema* Hua, *P*. *kingianum* was later accurately identified as a new species by Coll and Hemsl. The dried rhizome of *P*. *kingianum* has been used in Traditional Chinese Medicine for centuries. The polysaccharides, constituting the predominant and pivotal bioactive constituents of the rhizome of *P*. *kingianum*, are extracted at a rate ranging from 10 and 40% [6]. They exhibit a wide range of pharmacological effects encompassing immunomodulation [7], antioxidant activity [8], anti-inflammatory properties [9], as well as potential benefits for diabetes and metabolic syndrome [10]. However, it is noteworthy that there is limited literature available on the comprehensive structural analysis of polysaccharides in the genus Polygonatum. Previous studies have indicated that → 1)-β-D-Fru*f*-(2 → , → 1)-β-D-Man*p*-(4 → are the predominant sugar residues in *P*. *sibiricum* polysaccharides from Lueyang, Shaanxi [11], whereas → 1)-β-D-Gal*p*-(4 → , β-D-Gal*p*-(1 → , → 1)-α-L-Ara*f*-(5 → represent the major sugar residues in *P*. *sibiricum* polysaccharides from Anhui [12]. Regarding the *P*. *kingianum* polysaccharides, a complete polysaccharide from Puer, Yunnan, has been recently elucidated [13]. This polysaccharide consisted of monosaccharides including Fru, Glc, and Gal, and was characterized by a linear backbone with β-D-Fru*f*-(2 → serving as the sole side chain. These findings suggested that the variations in species and origins can lead to structural disparities in polysaccharides of the genus Polygonatum. However, given that the fine structure of the polysaccharide can influence its pharmacological effects, it is imperative to elucidate the complete polysaccharide structures from various origins of *P*. *kingianum* and to evaluate their activities.

Therefore, in this study, a novel polysaccharide, PKP1, was isolated from *P*. *kingianum* through a series of consecutive column chromatography. The structure of PKP1 was initially characterized using HPGPC, FT-IR, HPLC, and GC-MS, and further elucidated through NMR analysis. Additionally, the PKP1's anti-inflammatory activity and potential for gut microbiota regulation were also evaluated.

## Materials and methods

### Materials and chemicals

*P*. *kingianum* samples were collected from Jianshui, Honghe Hani and Yi Autonomous Prefecture, Yunnan Province, China. Cellulose DE-52 and Sephadex G-50 were procured from Beijing RuiDa HengHui Science and Technology Development Co., Ltd. Dextran standards were bought from Agilent Technologies, Inc. Monosaccharide standards: Man, Rha, Glc A, Gal A, Glc, Gal, Xyl, Ara, and Fru were obtained from Shanghai yuanye Bio-Technology Co., Ltd. CCK-8 reagent was purchased from APE × Bio. IL-6 and TNF-α kits were acquired from Elabscience Biotechnology Co., Ltd. C57BL/6 mice in SPF level were sourced from Liaoning Changsheng Biotechnology Co., Ltd. DMSO (anhydrous solvent grade) was obtained from Shanghai Aladdin Biochemical Technology Co., Ltd. Trifluoroacetic acid (TFA) and 1-phenyl-3-methyl-5-pyrazolone (PMP) were procured from Shanghai Macklin Biochemical Technology Co., Ltd. Iodomethane was bought from Anhui Senrise Technologies Co., Ltd. Chloroform was sourced from Tianjin Damao chemical reagent factory. All other chemicals used were of analytical grade.

### Extraction and purification of polysaccharides

Several rhizomes of *P*. *kingianum* were collected, dried, and pulverized. The powder was mixed with an 85% ethanol solution at the rate of 1:8 (w/v), soaked for 24 h, and subsequently dried. The dried powder was mixed with distilled water at a rate of 1:20 (w/v) and extracted at 65 ℃ for 2 h, repeated twice. The filtrates were combined and concentrated before adding four times (v/v) anhydrous ethanol and leaving it overnight. The precipitates were lyophilized to obtain crude polysaccharides. A Sevag reagent (chloroform: n-butanol = 4:1) in a ratio of 1/4 (v/v) was added to the crude polysaccharide solution, followed by centrifugation to collect the supernatant. This step was repeated until no flocculation occurred between the two phases. Subsequently, activated carbon (4%) was added to the filtrate and adsorbed for 1 h at 35 ℃; thereafter, the filtrate was dialyzed (cut-off molecular weight 3500 Da) for 48 h. Finally, *P*. *kingianum* polysaccharides (PKP) were obtained after alcohol precipitation and lyophilization.

The PKP sample was dissolved in distilled water, followed by centrifugation. The resulting supernatant was then applied onto a Cellulose DE-52 column. The column was eluted using distilled water and NaCl solutions with 0.05 M and 0.1–0.5 M, respectively, at a flow rate of 1 mL per minute. The eluate fractions were collected in tubes containing 10 mL each, and the polysaccharide content in the eluate was determined using the phenol–sulfuric acid method. The distilled water fraction was further purified using a Sephadex G-50 column. The column was eluted with distilled water at 0.5 mL per minute, and the eluate was collected in tubes containing 4 mL each. The polysaccharide content in the eluate was determined using the phenol–sulfuric acid method. Following alcohol precipitation and lyophilization, the obtained polysaccharide was designated as PKP1.

### Structural characterization of PKP1

#### SEM analysis

The PKP1 sample was sputtered with a thin layer of gold using an ion sputter coater. Subsequently, the morphology was observed using a scanning electron microscope (SEM, FlexSEM 1000, HITACHI, Japan) under high vacuum and 10 kV accelerating voltage conditions, with image magnifications of 200 and 1000 times.

#### Molecular weight and homogeneity analysis

The PKP1 was dissolved in distilled water to achieve a final concentration of 0.5% (w/v) and subsequently filtered through a 0.22 μm aqueous filter. Subsequently, it was analyzed using high-performance gel permeation chromatography (HPGPC) (Agilent, USA) equipped with a refractive index detector (RID) and a PL aquagel-OH 20 T column (7.8 mm × 300 mm, 9 μm). The mobile phase was distilled water at a flow rate of 0.5 mL/min with an injection volume of 5 µL. The molecular weight of PKP1 was calculated by determining a standard curve (Y = − 0.2519X + 12.583 [R^2^ = 0.9989]) utilizing dextran standards of known molecular weight (Mw) (180, 540, 5000, 10000, 20000 Da).

#### FT-IR spectroscopy analysis

The PKP1 sample weighing 2 mg was finely ground with 100 mg of KBr powder and subsequently pressed into a 1.0 mm slice. The fourier transform infrared spectroscopy (FT-IR) spectra were measured using FT-IR spectroscopy (6700, Thermo Fisher Scientific, USA) within the frequency range of 4000–400 cm^−1^.

#### Thermal analysis

The thermogravimetric analysis (TGA) of PKP1 was conducted using a thermogravimetric analyzer (Q50, TA, USA). A precisely measured 10 mg of PKP1 was subjected to a temperature ramp from 35 °C to 900 °C at a rate of 10 °C/min under a nitrogen atmosphere as the carrier gas.

#### Monosaccharide composition analysis

The monosaccharide composition was measured using two selected methods. Pre-column 1-phenyl-3-methyl-5-pyrazolone derivatization high-performance liquid chromatography (PMP-HPLC) was used to determine the monosaccharides other than Fru. Briefly, PKP1 was hydrolyzed with 2 M trifluoroacetic acid (TFA) at 120 ℃ for 4 h, followed by drying with a nitrogen flow and redissolving. The hydrolysis acid solution was then mixed with 0.3 M NaOH solution and 0.5 M PMP solution for derivatization at 70 ℃ for 1 h. The pH was adjusted using 0.3 M HCl, and chloroform extraction was utilized as the test solution. Monosaccharide standards were treated with the same derivatization method to obtain the standard solutions. Subsequently, they were analyzed using an HPLC (Waters, USA) equipped with a photo-diode array (PDA) detector and a YMC-Pack Pro C18 RS capillary column (4.6 mm × 250 mm, 5 μm). The mobile phase was acetonitrile-0.02 M ammonium acetate solution (20:80) at a flow rate of 1 mL/min with an injection volume of 20 µL.

Another method was used to determine Fru. PKP1 was hydrolyzed in 0.05 M TFA at 85 ℃ for 0.5 h, followed by drying with a nitrogen flow and redissolving. Monosaccharide standards were dissolved in distilled water and filtered to obtain the standard solutions. It was analyzed using HPLC (SHIMADZU, Japan) equipped with a refractive index detector (RID) and an Aminex HPX-87P sugar analyzing column (7.8 mm × 300 mm, 9 μm). The mobile phase was distilled water at a flow rate of 1 mL/min with an injection volume of 20 µL.

#### Methylation analysis

Methylation analysis was performed according to previously reported methods with slight modifications [14]. 10 mg of PKP1 was dissolved with 50 mg of NaH in anhydrous DMSO and stirred under nitrogen protection for 12 h. Subsequently, 2 mL of pre-cooled iodomethane was slowly added dropwise, and the reaction proceeded in the darkness for 2 h. This process was repeated three times, and the reaction was terminated by adding 3 mL of distilled water. The resulting solution was extracted with chloroform and dried using a nitrogen flow. After complete methylation, the product was hydrolyzed with 2 mL of 1 M TFA at 90 °C for 150 min. Then, 2 mL of 2 M ammonia water and excess NaBH_4_ were added and left overnight, and neutralized with 50% acetic acid to pH 6–7, then dried with nitrogen flow. 2 mL of acetic anhydride and 2 mL of pyridine acetylation were added and reacted at 90 ℃ for 2 h. Finally, the reaction was terminated by adding 2 mL of distilled water, followed by extraction with chloroform, removal of water using anhydrous Na_2_SO_4_, filtration through a 0.22 μm filter, and subsequent gas chromatograph-mass spectroscopy (GC-MS) analysis.

It was analyzed using a high-resolution orbitrap GC mass spectrometer (Exactive GC, Thermo Fisher Scientific, USA) equipped with a positive electron ionization (EI) source and an HP-5 capillary column (30 m × 0.25 mm, 0.25 μm). High-purity nitrogen was used as the carrier gas, the injection volume was 1 μL, the split ratio was 10:1, and the temperature of the ion source and the quadrupole was 230 ℃. The analytes were detected in full scan mode within the mass scan range (*m/z*) of 30–600. The temperature increase program consisted of an initial column oven temperature of 140 °C for 2 min, followed by an increase to 230 °C at a rate of 3 °C per minute, and holding at this temperature for another 3 min.

#### NMR analysis

The PKP1 was dissolved in D_2_O and subjected to repeated lyophilization. Subsequently, 80 mg of lyophilized PKP1 was dissolved in 1 mL of D_2_O. The 1D and 2D nuclear magnetic resonance spectroscopy (NMR) spectra were determined using a 600 MHz NMR spectrometer (JNM-ECZ600R/S1, JEOL, Japan). The 1D NMR included ^1^H NMR and ^13^C NMR, and the 2D NMR included ^1^H-^1^H correlation spectroscopy (^1^H-^1^H COSY), heteronuclear single-quantum correlation (HSQC), and heteronuclear multiple bond correlation (HMBC).

### Anti-inflammatory activity

#### Determination of cell viability

The cell viability of PKP1 and lipopolysaccharide (LPS) on RAW264.7 macrophages was evaluated using the CCK-8 assay. Briefly, RAW264.7 macrophages were seeded into 96-well plates (5 × 10^3^ cells/well) and incubated for 24 h. Subsequently, the cells were treated with various concentrations of PKP1 along with 1 μg/mL LPS for 24 h. After removing the supernatant, the CCK-8 reagent was added and incubated for 2 h. Finally, the absorbance at 450 nm was measured.

#### Measurement of inflammatory factors

RAW264.7 macrophages were seeded into 24-well plates (5 × 10^4^ cells/well), and the incubation and treatment procedures were performed as described above. The supernatants were collected at the end of the experiment and centrifuged at 1000 × *g* for 20 min. NO levels were determined using Griess reagent, and IL-6 and TNF-α levels were measured using an enzyme-linked immunosorbent assay kit following the manufacturer's instructions.

### Regulation of gut microbial structure and function in vitro

#### Diabetic mice and fecal samples

Male C57BL/6 mice (20–25 g) were purchased from the SPF laboratory animal center of Liaoning Changsheng Biotechnology Co., Ltd. (Benxi, China). High-fat diet (45% energy from fat, D12451) was purchased from SYSE Biotechnology Co., Ltd. (Changzhou, China). The experimental procedures involving animals were conducted following the International Ethical Guidelines and approved by the Institutional Animal Care Committee.

The prediabetic mice model was established according to our previous method with modifications [15, 16]. Briefly, C57BL/6 mice were fed a high-fat diet supplemented with 3% sucrose for 4 weeks to induce insulin resistance. Subsequently, the mice received 50 mg/kg streptozotocin (STZ) dissolved in 0.1 M citrate buffer (pH 4.2–4.5) via intraperitoneal injection. Oral glucose tolerance tests (OGTT) were performed on days 7, 14, and 21 post-injection, with plasma glucose levels measured at 0 and 120 min after a 2 g/kg oral glucose load using a Roche glucometer. Mice meeting prediabetes criteria (fasting plasma glucose (FPG): 6.0–7.0 mM and/or 2 h postprandial glucose (2h-PG): 7.8–11.1 mM) were selected as prediabetic models (n = 8). Normoglycemic littermates (FPG 3.2–6.0 mM; 2h-PG: 3.2–7.8 mM) served as controls (n = 8). Fecal samples from individual mice in the model and control groups were pooled separately, treated with PBS containing 20% glycerol, and stored at – 80 ℃ for subsequent gut microbiota culture experiments.

#### Culture of gut microbiota in vitro

The gut microbiota was cultured in vitro using our previously established method [17]. Based on previous studies demonstrating that a polysaccharide dosage of 200 mg/kg exhibited hypoglycemic effects in prediabetic mice [16]. We calculated the corresponding in vitro concentrations. Under these conditions, the gavage concentration of polysaccharide was approximately 0.5–1% (w/v), leading us to designate two experimental concentrations (0.5% and 1%, w/v). Two concentrations of PKP1 were added to the anaerobic culture medium, followed by inoculation with 1% (w/v) normal/model fecal slurry in each group. The cultures were incubated at 37 ℃ for 24 h on a shaker. After centrifugation, the supernatant and mycelium were stored at − 20 ℃ for subsequent analysis.

#### Microbial diversity analysis of the gut microbiota

The mycelia obtained from culture in vitro were entrusted to Hangzhou Guhe Information Technology Co., Ltd. (Hangzhou, China) for diversity analysis. Briefly, upon receipt of the samples, the total DNA was extracted, followed by a rigorous quality control assessment. Subsequently, PCR amplification was performed on the qualified samples. Electrophoresis was then carried out on the amplified products to ensure their quality and consistency with positive and negative controls from the same batch. The libraries that passed these tests were subjected to high-throughput sequencing using the Illumina NovaSeq platform. The sequencing data obtained were utilized for species identification analysis.

#### Determination of short-chain fatty acids

The short-chain fatty acids (SCFAs) in the supernatants were determined using our previously established method [17]. Briefly, 1 mL of supernatant and the solution of standards (acetic acid, propionic acid, isobutyric acid, and butyric acid) at various concentrations were thoroughly mixed with excess ammonium persulfate. Subsequently, 1 mL of anhydrous ethanol was added, and the mixture was vortexed and oscillated for 10 s. After centrifugation at 10,000 × *g* for 5 min, the resulting supernatant was collected for analysis. It was analyzed using a GC (SHIMADZU, Japan) equipped with a flame ionization detector (FID) and an FFAP capillary column (30 m × 0.25 mm, 0.25 μm). High-purity nitrogen was used as the carrier gas, the injection volume was 1 μL, the split ratio was 1:10, the flow rate was 2.96 mL/min, and the temperature of the injector as well as the detector was 250 ℃. The temperature increase program consisted of initially holding the temperature at 80 °C for 2 min, followed by an increase to 180 °C at a rate of 10 °C per minute, and holding at this temperature for another 3 min.

### Statistical analysis

The statistical analysis was performed using SPSS 23.0 software. Statistical differences between groups were assessed through one-way analysis of variance (ANOVA) followed by Tukey's *post-hoc* test, with a p-value of < 0.05 considered statistically significant. All data were expressed as mean ± standard deviation (SD).

## Results and discussion

### Isolation and purification of PKP1

**Fig. 1:**
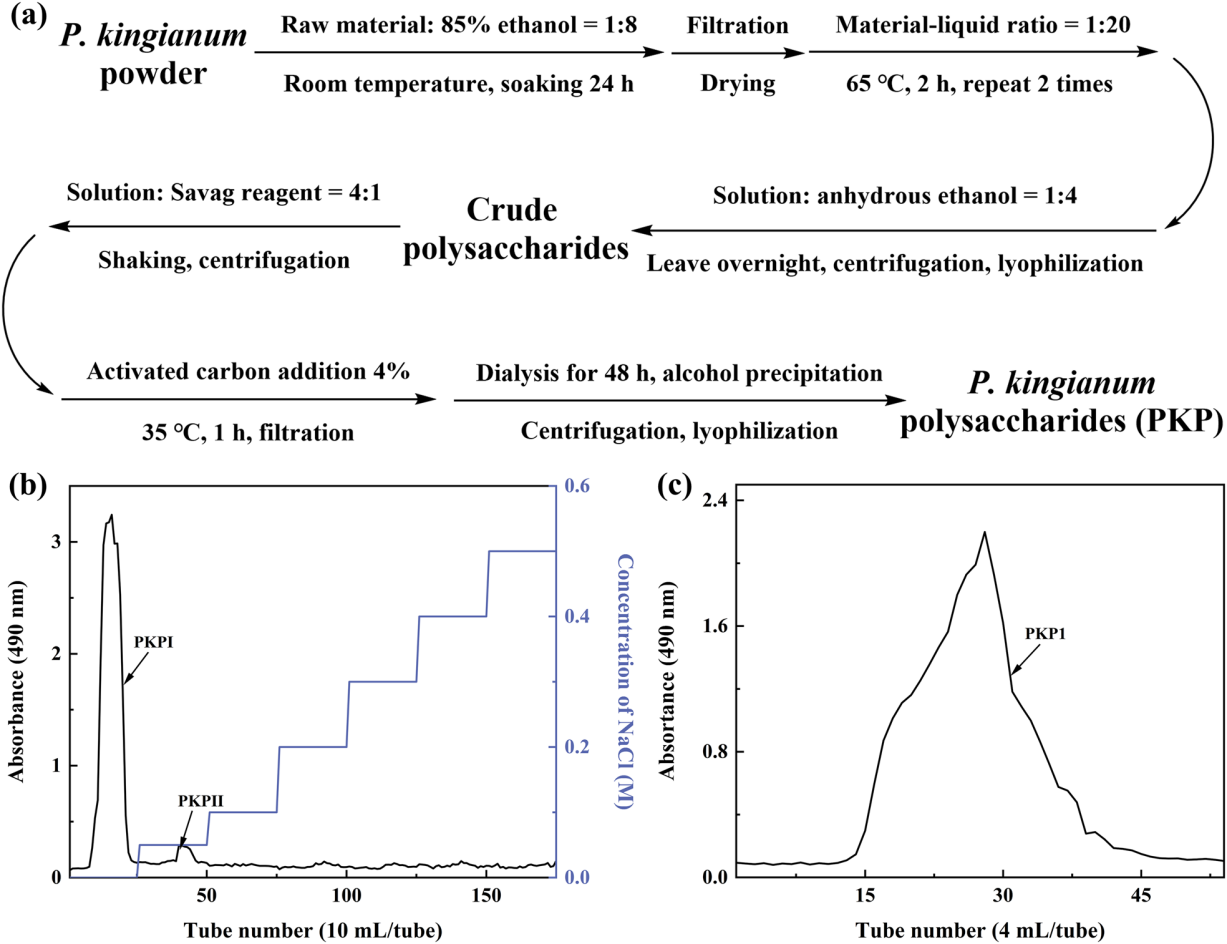
**a** Extraction process of *P*. *kingianum* polysaccharides. **b** Cellulose DE-52 column chromatography. **c** Sephadex G-50 column chromatography

*P*. *kingianum* underwent a series of processes, including lipid removal, aqueous extraction, protein removal, depigmentation, dialysis, alcohol precipitation, and lyophilization to obtain *P*. *kingianum* polysaccharides (PKP), as depicted in Fig. 1a. During this process, the yield of PKP from *P*. *kingianum* was 4.3%, with a total polysaccharide recovery rate of 29.3%. PKP was initially purified using a Cellulose DE-52 column. As shown in Fig. 1b, it was observed that PKP mainly consisted of two components, which were subsequently named PKPI and PKPII. Due to its significantly higher content compared to PKPII, PKPI was further purified using a Sephadex G-50 column (Fig. 1c). This purification step resulted in a single peak, which was designated as PKP1. Since Fru was the dominant component in the monosaccharide analysis, Fru was used as the standard to develop a calibration curve [18] and as shown in Fig. S1. The purity of PKP1 was as high as 96%, meeting the requirements of the subsequent structural characterization and analysis.

### Structural characterization of PKP1

#### SEM analysis of PKP1

**Fig. 2:**
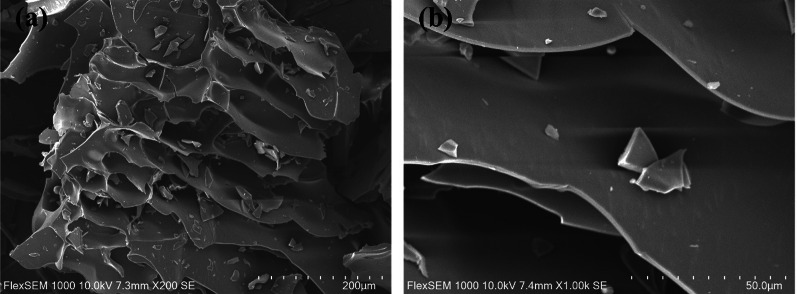
SEM image of PKP1. **a** 200 × . **b** 1000 ×

The PKP1 was examined using SEM, and the data were shown in Fig. 2. It could be observed that PKP1 exhibited a honeycomb structure with a relatively compact and smooth surface. Furthermore, it significantly increases its specific surface area due to the porous structure. Previous studies have indicated that the morphology of polysaccharides indirectly affects the properties of polysaccharides [19, 20]. The presence of pores and grooves on the surface of polysaccharides increased their specific surface area, exposing more hydroxyl groups that could form hydrogen bonds with water molecules, thus improving solubility. Therefore, the SEM data suggested better solubility for PKP1, consistent with our observation that it was easily dissolved in distilled water during experiments.

#### Molecular weight and homogeneity of PKP1

**Fig. 3:**
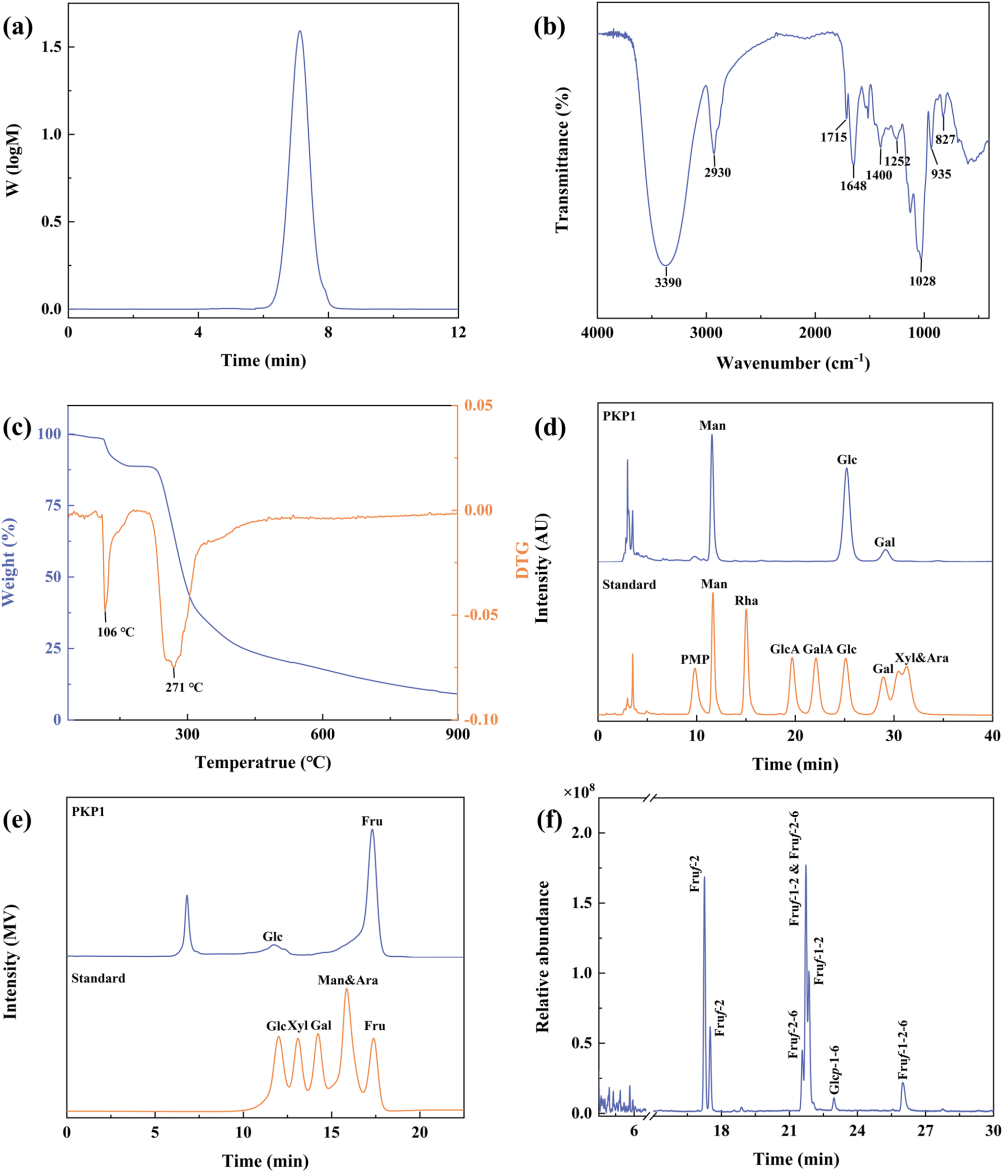
**a** HPGPC curve of PKP1. **b** FT-IR spectra of PKP1. **c** TG and DTG curves of PKP1. **d** HPLC of PKP1 monosaccharide composition (without Fru). **e** HPLC of PKP1 monosaccharide composition (with Fru). **f** GC-MS profile of PKP1

The molecular weight (Mw) and the homogeneity of PKP1 were measured using HPGPC, and the data were shown in Fig. 3a. It is evident from the HPGPC profile that PKP1 exhibited a single and symmetrical peak. The Mw of PKP1 was measured to be 5.3 × 10^3^ Da by employing the standard regression equation. In comparison with previously reported Mw of *P*. *kingianum* polysaccharides, such as 14.05 × 10^3^ Da and 8.7 × 10^3^ Da [20, 21], PKP1 displayed a lower Mw, which was speculated to result in better solubility. Furthermore, the polymer dispersity index (PDI) of PKP1, which was defined as the ratio of Mw/Mn, was calculated to be 1.20. A smaller PDI value suggested a narrower Mw distribution and better homogeneity [22], thus meeting the requirements for the subsequent structural analysis.

#### FT-IR spectral analysis of PKP1

The FT-IR data in Fig. 3b indicated that the peaks observed at 3390 cm^−1^, 2930 cm^−1^, and 1028 cm^−1^ corresponded to the stretching vibrations of O–H, C–H, and C–O bonds, respectively, representing characteristic absorption bands of classical polysaccharides. The peak observed at 1715 cm^−1^ was attributed to the stretching vibration of esterified carboxylate [23], while the peak at 1648 cm^−1^ originated from bound water within the polysaccharide structure [24]. The peak at 1400 cm^−1^ corresponded to the bending vibrations of C–H bonds [25], whereas the appearance of a peak at 1252 cm^−1^ indicated the vibrational modes associated with the *O*-acetyl group (–OAc). It was noteworthy that the absorption peaks at 935 cm^−1^ and 827 cm^−1^ were distinctive features indicative of β-D-Fru [23], which was consistent with monosaccharide composition results.

#### Thermal analysis of PKP1

The thermal stability of PKP1 was characterized using thermogravimetric analysis (TGA). The thermogravimetry (TG) curve, which depicted the weight change pattern of the sample during the programmed heating, and the derivative thermogravimetry (DTG) curve, which indicated significant changes in the rate of sample mass loss at specific temperatures, were shown in Fig. 3c. Consequently, the TG curve of PKP1 exhibited a three-step degradation pattern, with two distinct mass losses occurring around 106 ℃ and 271 ℃, as revealed by the DTG curve. These were attributed to the loss of bound water in polysaccharides and depolymerization of the polysaccharide structure, respectively [26]. Consequently, these findings indicated that PKP1 exhibited thermal stability below 106 ℃.

#### Monosaccharide composition analysis of PKP1

Previous studies have indicated that under conditions of high temperature and strong acidity, Fru readily undergoes conversion into Man and Glc. Moreover, due to its inherent structural properties, PMP becomes undetectable following PMP derivatization. Although reducing acid concentration can prevent Fru conversion, this compromises the complete hydrolysis of other monosaccharides [23, 27]. Therefore, the monosaccharide composition of PKP1 was determined using two HPLC methods: PMP-HPLC-PDA, characterized by high-concentration TFA and prolonged heating at high temperatures, and HPLC-RID, characterized by low-concentration TFA and a lower-temperature heating pretreatment. As anticipated, chromatographic analysis using PMP-HPLC-PDA (Fig. 3d) detected only Man, Glc, and trace amounts of Gal. In contrast, HPLC-RID (Fig. 3e) confirmed that the monosaccharides of this polysaccharide primarily consist of Fru and Glc, indicating that the Man detected via PMP-HPLC-PDA mainly originates from the conversion of Fru. Considering the specificity of Fru's presence, the actual Fru/Glc content ratio was subsequently determined by NMR analysis rather than by these two HPLC methods [11, 28]. Notably distinct differences were observed when comparing the monosaccharide compositions of *P*. *kingianum* polysaccharides reported in previous studies [14, 20, 21]. For instance, *P*. *kingianum* derived from Dali, Yunnan, exhibited a composition comprising Glc, Man, Gal A, Gal, Glc A, and Ara, whereas *P*. *kingianum* obtained from Ailao Mountain, Yunnan, consisted primarily of Fru, Gal, Gal A, and Glc.

#### Methylation analysis of PKP1

Methylation of the polysaccharides, followed by hydrolysis and acetylation, combined with GC-MS analysis of the resulting products, proved to be a highly effective method for determining the sugar residues present in polysaccharides [14]. The GC spectrum was shown in Fig. 3f, while fragment data and the molar ratio of each sugar residue obtained from integrating the methylation product in GC were summarized in Table 1. The corresponding MS spectra were shown in the supporting information in Fig. S2. The fragment data in GC-MS indicated that PKP1 contained five distinct types of sugar residues, which were β-D-Fru*f*-(2 → , → 1)-β-D-Fru*f*-(2 → , → 2)-β-D-Fru*f*-(6 → , → 1)-α-D-Glc*p*-(6 → and → 1,2)-β-D-Fru*f*-(6 → , respectively [11, 29]. Among these residues, three sugar residues, including β-D-Fru*f*-(2 → , → 2)-β-D-Fru*f*-(6 → and → 1)-β-D-Fru*f*-(2 → , accounted for 72.16% of the molar ratio, suggesting their prominence as the major sugar residues. It is worth noting that the Man was not detected, confirming the results in monosaccharide composition analysis. Thus, PKP1 might be a novel Fru-dominated neutral polysaccharide. It has been reported that the reduction of methylated D-Fru resulted in the formation of D-mannitol and D-glucitol derivatives [28, 30], and Fru was susceptible to transformation during acidolysis as mentioned in Sect. 3.2.3; hence, their ratios could not be determined but will be resolved using NMR subsequently.

**Table 1:** Methylation mass spectrometry fragmentation analysis of PKP1

RT (min)	Methylated sugar	Major mass fragments (m/z)	Type of linkage	Molar ratio
17.25	2,5-di-O-acetyl-1,3,4,6-tetra-O-methyl mannitol	71,85,87,101,129,145,161,205,245	Fru*f*-(2 →	25.77
17.50	2,5-di-O-acetyl-1,3,4,6-tetra-O-methyl glucitol	71,79,87,101,129,145,161,205,245	Fru*f*-(2 →	9.66
21.57	2,5,6-tri-O-acetyl-1,3,4-tri-O-methyl glucitol	79,87,99,129,145,161,189,205,233	→ 2)-Fru*f*-(6 →	7.14
21.73	1,2,5-tri-O-acetyl-3,4,6-tri-O-methyl mannitol & 2,5,6-tri-O-acetyl-1,3,4-tri-O-methyl mannitol	71,87,99,129,145,161,189,205,233	→ 1)-Fru*f*-(2 → & → 2)-Fru*f*-(6 →	29.59
21.86	1,2,5-tri-O-acetyl-3,4,6-tri-O-methyl glucitol	79,87,99,129,145,161,189,205,233	→ 1)-Fru*f*-(2 →	18.74
22.96	1,5,6-tri-O-acetyl-2,3,4-tri-O-methyl glucitol	87,99,101,117,129,161,189,203,233	→ 6)-Glc*p*-(1 →	1.93
25.99	1,2,5,6-tetra-O-acetyl-3,4-di-O-methyl mannitol & 1,2,5,6-tetra -O-acetyl-3,4-di-O-methyl glucitol	79,87,99,129,189,233	→ 1,2)-Fru*f*-(6 →	7.16

#### NMR analysis of PKP1

To comprehensively elucidate the complete structure, the use of 1D/2D NMR, along with the results of monosaccharide composition and methylation, was necessary.

**Fig. 4:**
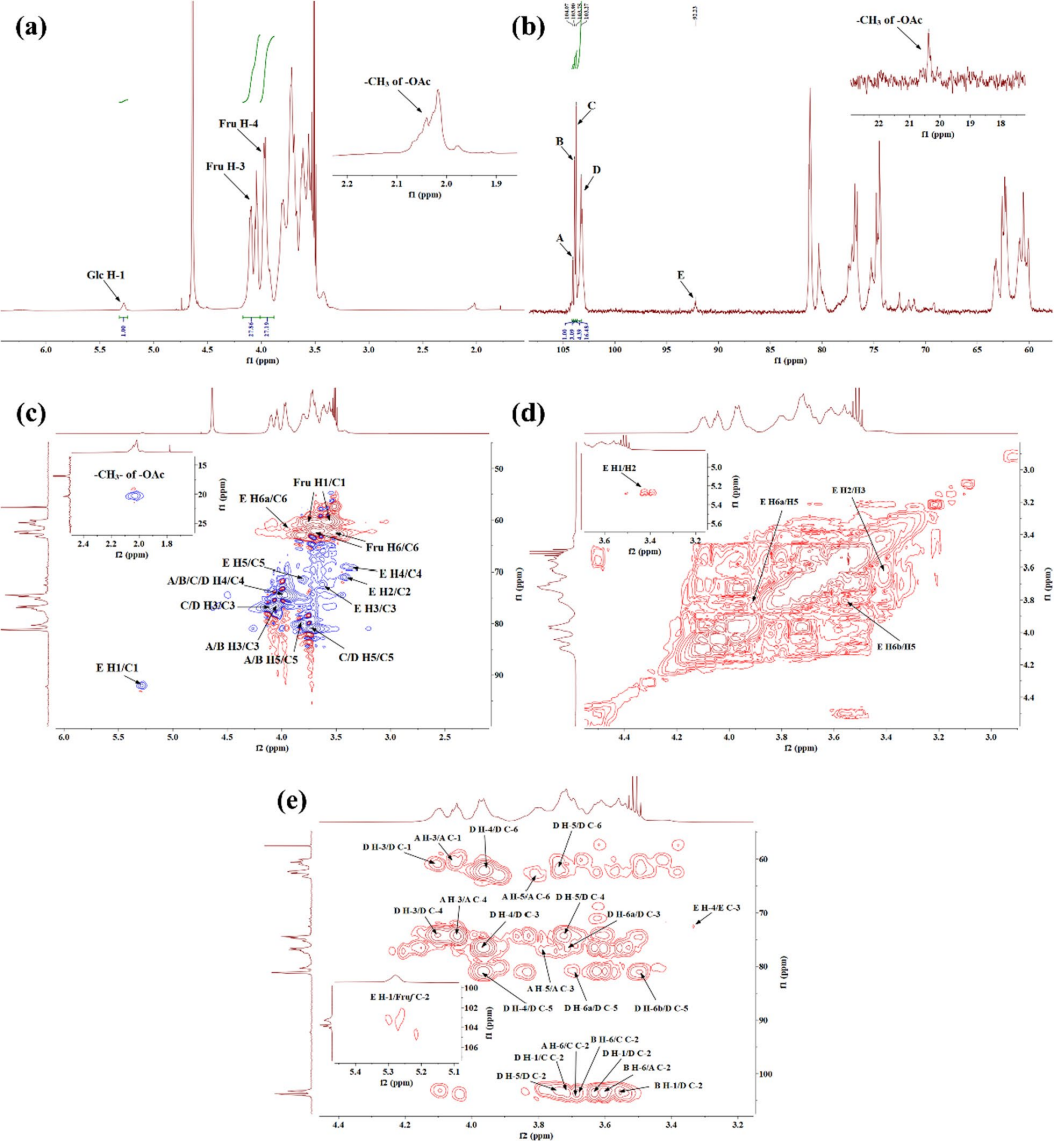
NMR spectrum of PKP1 (600 MHz, solvent D_2_O). **a** ^1^H NMR. **b** ^13^C NMR. **c** HSQC. **d** ^1^H-^1^H COSY. **e** HMBC

In the ^13^C NMR of PKP1 (Fig. 4b), two distinct sets of signals were observed in the anomeric carbon region at δ 90–110, indicating that PKP1 was mainly composed of two different types of monosaccharides. The results of monosaccharide composition and methylation indicated that PKP1 was predominantly composed of Fru. As ketoses lack anomeric hydrogen [28], no corresponding anomeric hydrogen was observed in the ^1^H NMR spectrum (Fig. 4a). Meanwhile, one set of the anomeric carbon signals at δ 104.07, δ 103.90, δ 103.75, and δ 103.27 exhibited significantly higher signal intensities and were consistent with the NMR characteristics of the carbon at 2-position in Fru*f*. Based on these findings along with previous studies [11, 31, 32], these singles were attributed to → 2)-β-D-Fru*f*-(6 → , → 1,2)-β-D-Fru*f*-(6 → , → β-D-Fru*f*-(2 → , and → 1)-β-D-Fru*f*-(2 → , and these sugar residues were named A, B, C, and D, respectively. Consequently, the anomeric carbon signal with lower intensity at δ 92.23 was attributed to α-configuration Glc*p* through comparison with published data [11, 29, 32]. Again, no obvious Man signals were detected.

In the ^1^H NMR spectrum, the signal at δ 5.28 was assigned to the anomeric hydrogen of Glc*p*, which was further confirmed by the HSQC and ^1^H-^1^H COSY data (Fig. 4c, d), illustrating its correlation with a carbon signal at δ 92.23. These findings were corroborated by methylation analysis, confirming the existence of a fifth sugar residue, → 1)-α-D-Glc*p*-(6 → [11, 29, 32], with substitution occurring at its 6-position in the neo-ketose form [32]. This sugar residue was designated as E. Furthermore, the obvious relationships among A, B, C, D, and E were observed, indicating that these sugar residues were the fundamental units of the PKP1. The chemical shift signals for both ^1^H and ^13^C of A, B, C, D, and E were assigned, as shown in Table 2.

**Table 2:** NMR signal attribution of PKP1 glycosidic bonds

Glycosidic bond	H1	H2	H3	H4	H5	H6
	C1	C2	C3	C4	C5	C6
A	3.61,3.56	–	4.04	3.96	3.81	3.69,3.62
	60.55	104.07	76.61	74.75	80.30	62.25
B	3.61,3.56	–	4.04	3.96	3.81	3.69,3.62
	60.55	103.90	76.61	74.75	80.30	62.25
C	3.61,3.56	–	4.10	3.98	3.73	3.69,3.54
	60.55	103.75	76.84	74.45	81.16	62.62
D	3.72,3.63	–	4.10	3.98	3.73	3.69,3.49
	60.55	103.27	76.84	74.45	81.16	62.23
E	5.28	3.42	3.63	3.33	3.82	3.92,3.59
	90.23	71.11	72.51	69.20	71.62	61.68

Due to the susceptibility of Fru to degradation under harsh conditions, the ratios of peak areas and molar abundances in monosaccharide composition and methylation analyses were not reflective of the true ratios. Therefore, NMR signal integration was used to address this issue. The ratio of A, B, C, and D was determined by integrating the anomeric carbon signal of Fru*f* at the 2-position in ^13^C NMR [11], resulting in a ratio of 1.00: 3.09: 4.39: 16.48, respectively. Additionally, the ratio of Fru*f* to Glc*p* was determined by integrating the signals of H-3 on Fru*f* and H-1 on Glc*p* in ^1^H NMR, yielding a ratio of 27.86 to 1.00. Consequently, an approximate ratio of the five sugar residues A: B: C: D: E was calculated as 1:3:5:18:1.

**Fig. 5:**
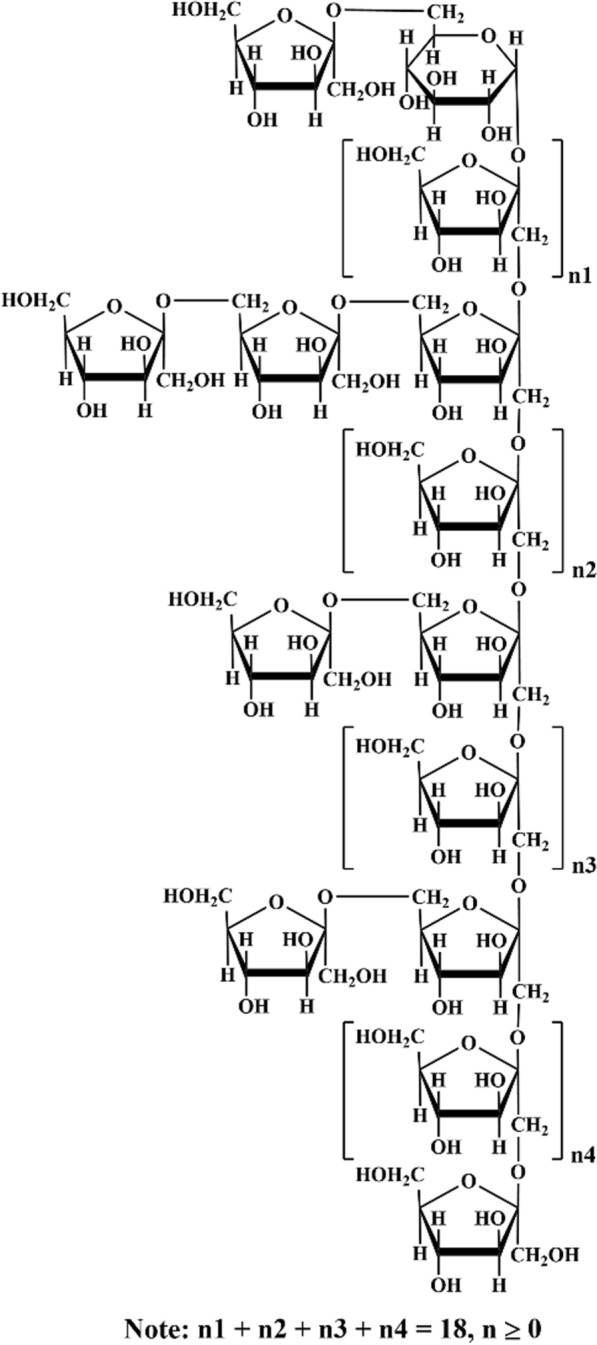
Proposed structure of PKP1

The linkages of the sugar residues were determined by HMBC, and the data were shown in Fig. 4e. There were obvious correlations between H-1 of sugar residue D (δ 3.72) and C-2 of sugar residue C (δ 103.75), as well as H-1 of sugar residue B (δ 3.56) and C-2 of sugar residue D (δ 103.27), indicating the presence of β-D-Fru*f*-(2 → 1)-β-D-Fru*f*-(2 → and → 1)-β-D-Fru*f*-(2 → 1)-β-D-Fru*f*-(2,6 → linkages, respectively. Additionally, the correlations between H-6 of sugar residues A and B (δ 3.69) and C-2 of sugar residue C (δ 103.75), along with the relationship between H-6 of sugar residue B (δ 3.62) and C-2 of sugar residue A (δ 104.07), indicated the existence of β-D-Fru*f*-(2 → 6)-β-D-Fru*f*-(2 → , β-D-Fru*f*-(2 → 6)-β-D-Fru*f*-(1,2 → and → 6)-β-D-Fru*f*-(2 → 6)-β-D-Fru*f*-(1,2 → linkages within PKP1 structure. Furthermore, the correlation between H-1 of sugar residue D (δ 3.63) and C-2 of the same sugar residue D (δ 103.27) suggested that the sugar chain was extended through a → 1)-β-D-Fru*f*-(2 → 1)-β-D-Fru*f*-(2 → linkage within sugar residue D itself. In addition, a signal cross peak was observed near δ 103 between H-1 of sugar residue E and Fru*f* C-2 in HMBC [33]. Combining the above 1D/2D NMR analyses with the monosaccharide composition, FT-IR, and methylation results, the structure link of PKP1 was finally elucidated, as shown in Fig. 5. Notably, the inferred structure contained 28 monosaccharides, which closely match the Mw of PKP1 (5.3 × 10^3^ Da) determined by HPGPC. The relatively small PDI value further supported that PKP1 might exist as a polymer formed with this glycan chain serving as an independent monomer.

Besides, there were still discernible weak signals, such as δ 1.90–2.10 in ^1^H NMR and δ 20.37 in ^13^C NMR. These signals exhibited characteristic features of –OAc groups and were further confirmed by the HSQC data [34]. However, no signal peaks associated with these signals were detected in NMR spectra, thus indicating that they could be attributed to impurities.

Therefore, in our study, a novel polysaccharide with a small PDI value was purified and elucidated. The main chain of this polysaccharide consisted of β-D-Fru*f*-(2 → , → 1,2)-β-D-Fru*f*-(6 → , → 1)-β-D-Fru*f*-(2 → and → 1)-α-D-Glc*p*-(6 → , while the side chain contained β-D-Fru*f*-(2 → and → 2)-β-D-Fru*f*-(6 → . Notably, these structural features differed from the previously reported *P*. *kingianum* polysaccharides, thereby expanding the structural diversity within this study.

### Anti-inflammatory activity

#### Effects of PKP1 and LPS on cell viability in LPS-induced RAW264.7 macrophages

**Fig. 6:**
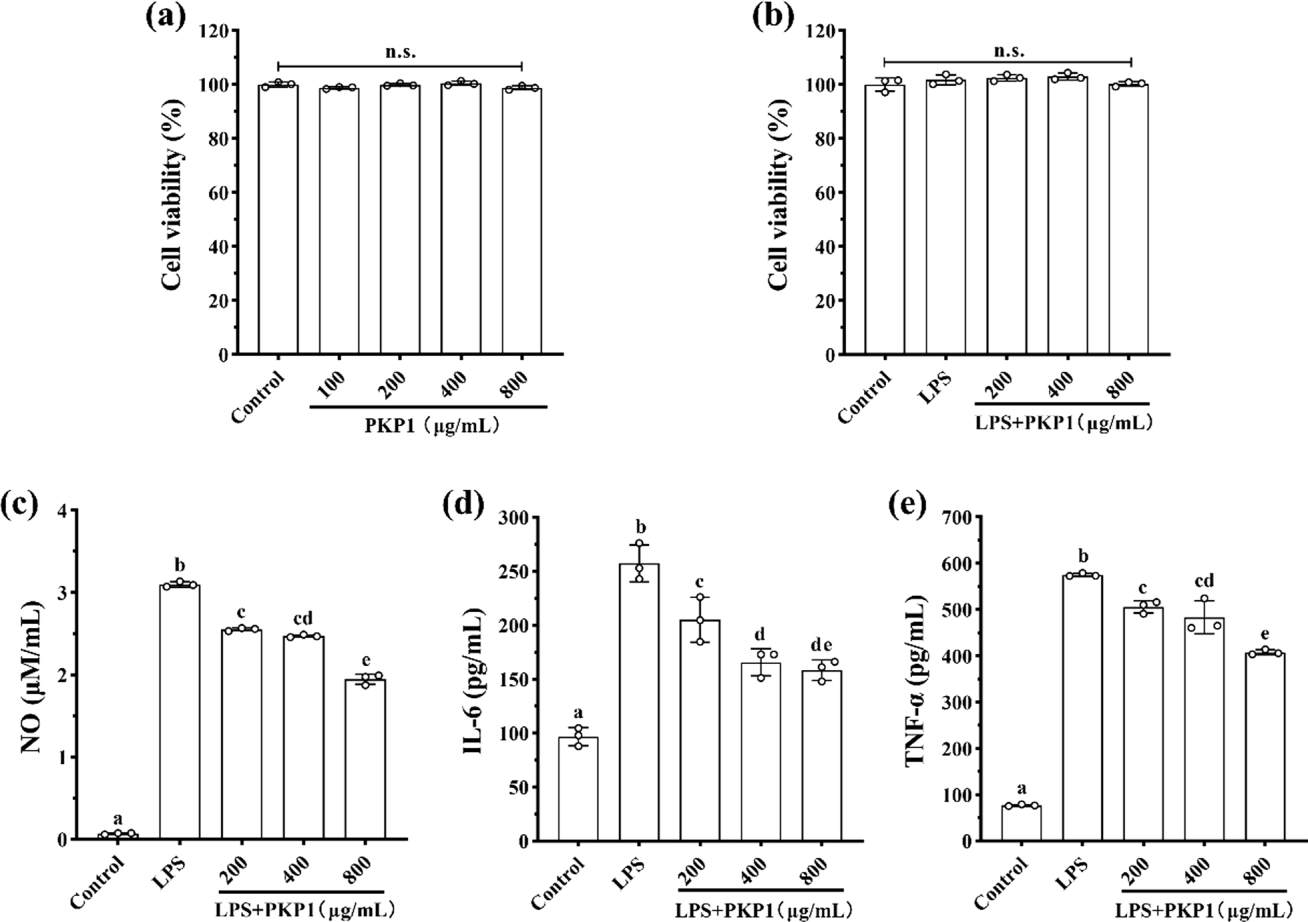
Effects of PKP1 on LPS-induced RAW264.7 macrophages. **a** Effects of PKP1 on cell viability. **b** Effects of PKP1 + LPS on cell viability. **c** NO levels. **d** IL-6 levels. **e** TNF-α levels. Statistical differences between groups were evaluated using one-way ANOVA, followed by Tukeys post-hoc test, and a p-value < 0.05 was considered statistically significant. Values are shown as mean ± SD. Values sharing a common letter (**a**, **b**, **c**, **d**, and **e**) in each row indicate no statistical significance between each group

The anti-inflammatory activity of PKP1 was initially evaluated using LPS-induced RAW264.7 macrophages to assess its biological activities. Cell viability served as the basis for immune activation and as an indicator of drug cytotoxicity. As shown in Fig. 6a, PKP1 demonstrated over 98% cell viability within the concentration range of 100–800 μg/mL, exhibiting no significant variation among these concentrations compared to the normal group. Furthermore, as depicted in Fig. 6b, when combined with a 1 μg/mL LPS solution, PKP1 at concentrations of 200–800 μg/mL exhibited a slight increase in cell survival rate compared to the normal group. This observation indicated that the combined action of PKP1 with 1 μg/mL LPS on the RAW264.7 macrophages was non-cytotoxic, thereby enabling subsequent studies.

#### Effects of PKP1 on inflammatory factor levels in LPS-induced RAW264.7 macrophages

Inflammatory factors are one of the important factors inducing insulin resistance (IR). By alleviating the inflammatory response, the production of inflammatory factors can be inhibited, thereby reducing IR and indirectly improving glycolipid metabolism [35]. The effects of PKP1 on reducing the levels of inflammatory factors in LPS-induced RAW264.7 macrophages were measured, and the data were presented in Fig. 6c, d, and e. The levels of NO, IL-6, and TNF-α were significantly increased in LPS-induced RAW264.7 macrophages; however, their levels were significantly reduced upon administration of PKP1. Specifically, when the PKP1 concentration was 800 μg/mL, the levels of NO, IL-6, and TNF-α were reduced by 37.10%, 38.44%, and 29.08%, respectively, compared with the LPS group. These findings indicated the anti-inflammatory activities of PKP1.

### PKP1 regulates gut microbial structure and function in vitro

#### Effects of PKP1 on the structure of gut microbial in vitro

**Fig. 7:**
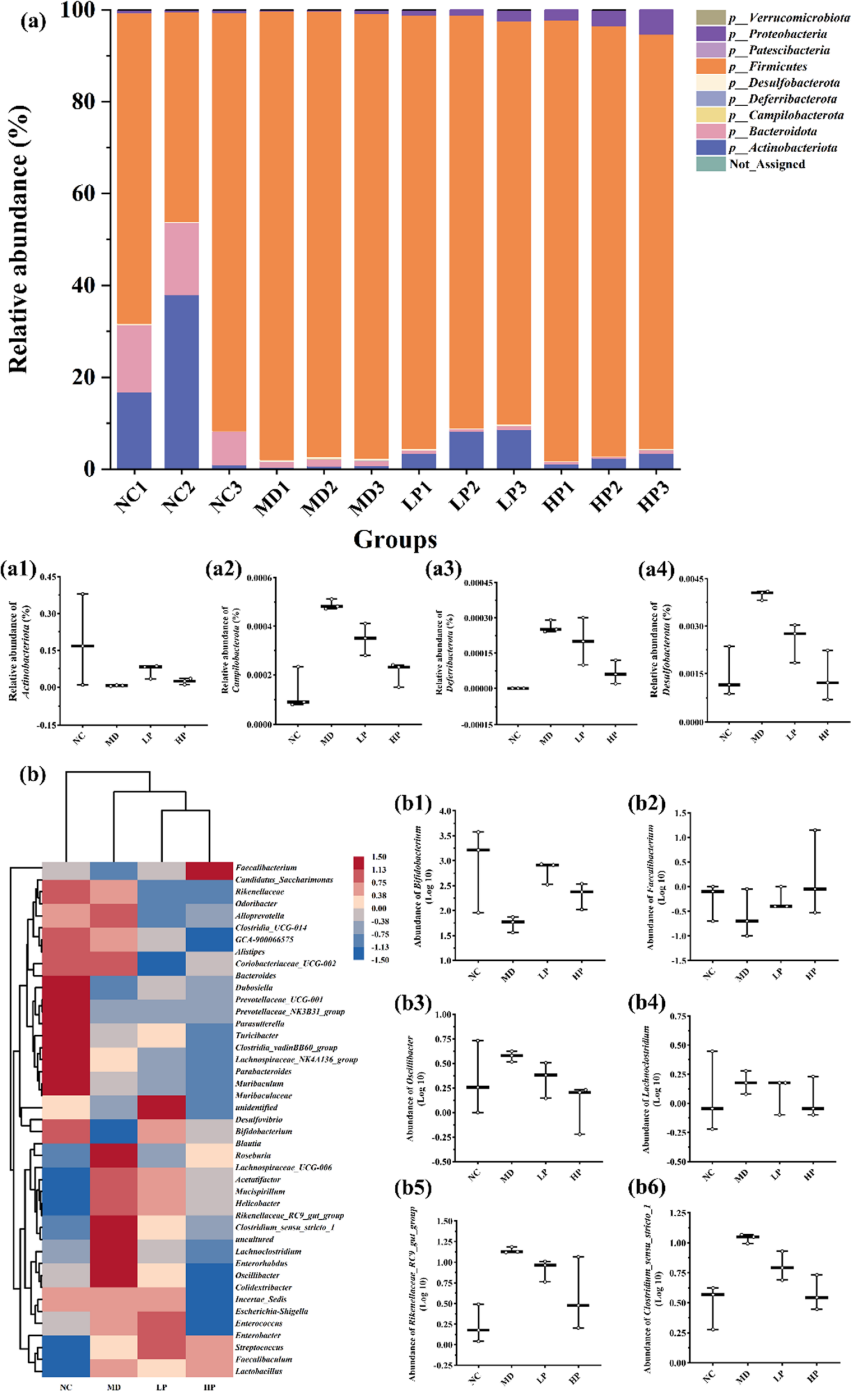
Effects of PKP1 on the structure of gut microbial in vitro. **a** Histogram of intergroup species composition at the phylum level. **a1-4** Relative abundance of phylum *Actinobacteriota*, *Campilobacterota*, *Deferribacterota*, *Desulfobacterota*. **b** Heatmap of intergroup species clustering at the genus level. **b1-6** Relative abundance of genus *Bifidobacterium*, *Faecalibacterium*, *Oscillibacter*, *Lachnoclostridium*, *Rikenellaceae_RC9_gut_group*, *Clostridium_sensu_stricto_1*

We subsequently assessed the impact of PKP1 on the regulation of gut microbiota in vitro. Utilizing our previously established in vitro gut microbiota model derived from diabetic mice [17], the effects of PKP1 on the structure of gut microbiota were also measured, as shown in Fig. 7. At the phylum level (Fig. 7a), the relative abundance of *Actinobacteriota* (Fig. 7a1), which was considered to play a crucial role in maintaining intestinal homeostasis [36], increased in the administered group, especially the LP group. Conversely, in both the LP group and HP group, the relative abundances of *Campilobacterota* (Fig. 7a2), *Deferribacterota* (Fig. 7a3), and *Desulfobacterota* (Fig. 7a4), which were correlated with abdominal pain, diarrhea, intestinal cell damage, and acute enteritis [37], all decreased. Previous studies have indicated that *Deferribacterota* and *Desulfobacterota* are positively correlated with body weight and lipid levels in high-fat diet model mice [38]. Therefore, our data hint at a potential effect of PKP1 on reducing body weight. At the genus level (Fig. 7b), the relative abundance of beneficial genera *Bifidobacterium* (Fig. 7b1) and *Faecalibacterium* (Fig. 7b2) recognized as probiotics for alleviating diabetes mellitus and metabolic syndrome [39, 40], increased in the administered group. In contrast, the abundance of harmful genera *Oscillibacter* (Fig. 7b3), *Lachnoclostridium* (Fig. 7b4), *Rikenellaceae_RC9_gut_group* (Fig. 7b5), and *Clostridium_sensu_stricto_1* (Fig. 7b6), which were positively correlated with diabetes [41–43], all decreased, especially the HP group. These findings indicated the beneficial effects of PKP1 on the treatment of diabetes mellitus.

#### Effects of PKP1 on metabolite SCFAs levels in vitro

**Fig. 8:**
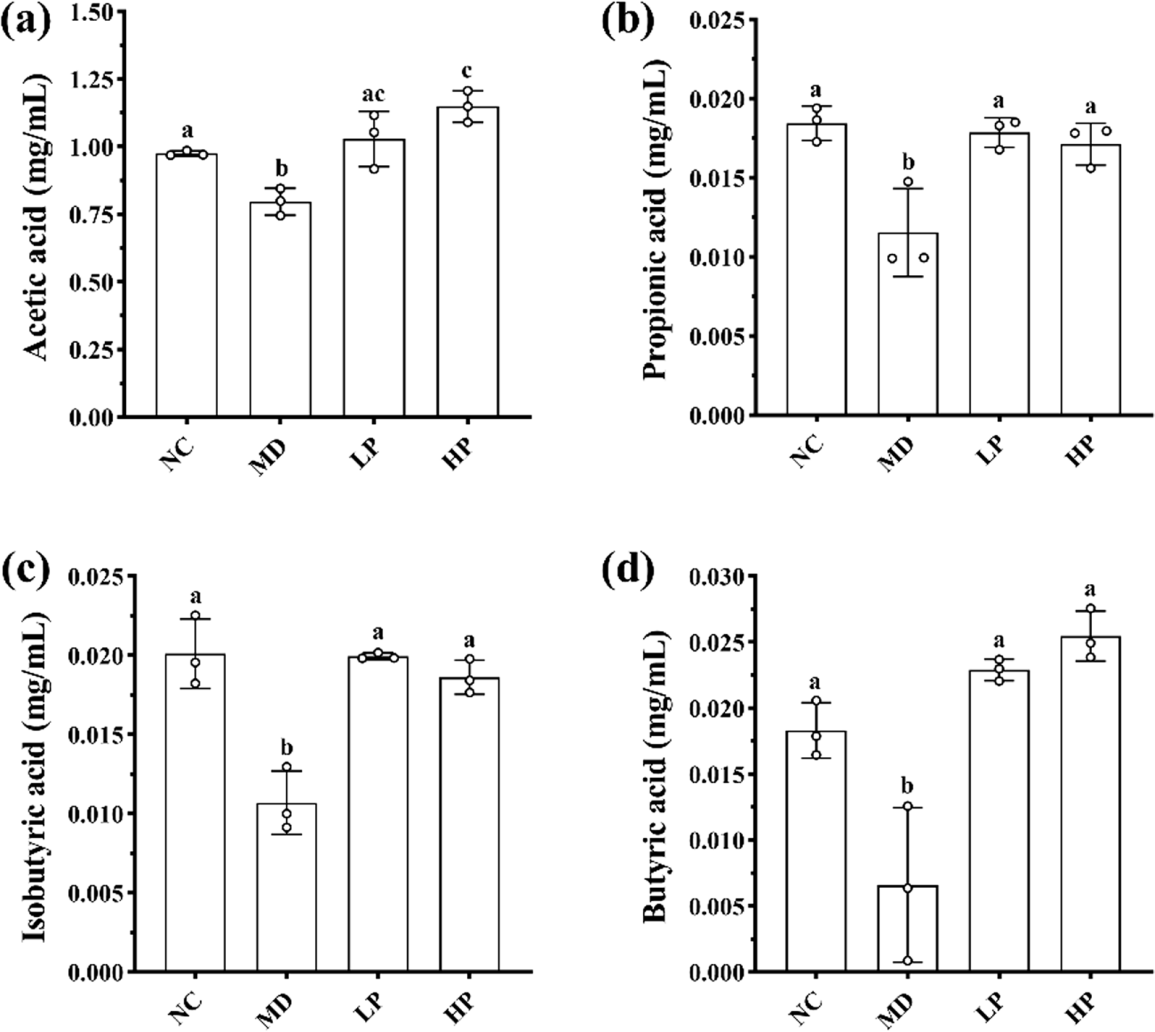
Effects of PKP1 on the levels of four SCFAs. **a** Acetic acid level. **b** Propionic acid level. **c** Isobutyric acid level. **d** Butyric acid level. Statistical differences between groups were evaluated using one-way ANOVA, followed by Tukey's post-hoc test, and a p-value < 0.05 was considered statistically significant. Values are shown as mean ± SD. Values sharing a common letter (**a**, **b**, and **c**) in each row indicate no statistical significance between each group

To further investigate the regulatory mechanism of PKP1 on gut microbiota [44], the SCFA levels in the diabetic mice gut microbiota were also measured, as depicted in Fig. 8. Consequently, the levels of all four types of SCFAs were significantly reduced in the MD group, while these SCFAs in both the LP group and HP group significantly increased following PKP1 intervention. Especially when PKP1 was added at 1%, the levels of acetic acid, propionic acid, isobutyric acid, and butyric acid were increased by 43.75%, 41.67%, 72.73%, and 278%, respectively, compared with the MD group (Table S1). It was worth noting that the levels of acetic acid and butyric acid increased in a dose-dependent manner. In the structural analysis of gut microbiota in vitro, the relative abundances of *Oscillibacter* and *Lachnoclostridium* were reduced, while the relative abundances of *Bifidobacterium* and *Faecalibacterium* increased after PKP1 administration. The former two gut bacteria exhibited negative correlations with SCFA levels, whereas the latter two demonstrated positive correlations. Therefore, our findings revealed that PKP1 increased the levels of SCFAs by regulating the gut microbiota composition.

## Conclusion

In this study, a polysaccharide from *P*. *kingianum* with a purity of 96% was purified using a multi-step process involving precipitation, ion exchange chromatography, and gel filtration chromatography. The resulting purified polysaccharide, designated as PKP1, mainly consisted of Fru and Glc with a molecular weight of 5.3 × 10^3^ Da and a PDI value of 1.20. Morphological and thermogravimetric analyses demonstrated the excellent solubility and thermal stability of PKP1. Methylation analysis revealed the presence of five sugar residues in PKP1, including → 2)-β-D-Fru*f*-(6 → , → 1,2)-β-D-Fru*f*-(6 → , β-D-Fru*f*-(2 → , → 1)-β-D-Fru*f*-(2 → and → 1)-α-D-Glc*p*-(6 → . The complete structure of PKP1 was elucidated using 1D/2D NMR, which differed from known polysaccharides, thus identifying it as a novel neutral polysaccharide. in vitro assays showed that PKP1 could significantly reduce the levels of inflammatory factors NO, IL-6, and TNF-α in LPS-induced RAW264.7 macrophages. Furthermore, PKP1 modulated gut microbiota by increasing the relative abundance of the beneficial genera *Bifidobacterium* and *Faecalibacterium*, while reducing the relative abundance of the harmful genera *Oscillibacter*, *Lachnoclostridium*, *Rikenellaceae_RC9_gut_group*, and *Clostridium_sensu_stricto_1*. Simultaneously, PKP1 increased the levels of SCFAs, including acetic acid, propionic acid, isobutyric acid, and butyric acid. Our study introduces a new polysaccharide from *P*. *kingianum* and provides valuable insights for the development of *P*. *kingianum* as a potential functional food supplement.

## Supplementary Information

Supplementary Material 1.

## Data Availability

The datasets used or analyzed during the current study are available from the corresponding author on reasonable request.

## Abbreviations

DTG: Derivative thermogravimetry
EI: Electron ionization
FID: Flame ionization detector
FPG: Fasting plasma glucose
FT-IR: Fourier transform infrared spectroscopy
GC–MS: Gas chromatography-mass spectrometry
^1^H-^1^H COSY: ^1^H-^1^H correlation spectroscopy
HMBC: Heteronuclear multiple bond correlation
HPGPC: High-performance gel permeation chromatography
HPLC: High-performance liquid chromatography
HSQC: Heteronuclear single-quantum correlation
IL-6: Interleukin-6
IR: Insulin resistance
LPS: Lipopolysaccharides
Mw: Molecular weight
NMR: Nuclear magnetic resonance spectroscopy
OGTT: Oral glucose tolerance test
PDA: Photo-diode array
PDI: Polymer dispersity index
PMP: 1-Phenyl-3-methyl-5-pyrazolone derivatization
*P. cyrtonema*: *Polygonatum* *cyrtonema* Hua
*P*. *kingianum*: *Polygonatum* *kingianum* Coll. et Hemsl.
*P*. *sibiricum*: *Polygonatum* *sibiricum* Red.
RID: Refractive index detector
SCFAs: Short-chain fatty acids
SEM: Scanning electron microscope
TFA: Trifluoroacetic acid
TGA: Thermogravimetric analysis
TNF-α: Tumor necrosis factor-α
