# Supplementary material for: A novel polysaccharide in Polygonatum kingianum: structure elucidation, the activities of anti-inflammatory and the regulation of gut microbiota in vitro
Source: Nat Prod Bioprospect. 2025 Sep 2;15(1):60. doi: 10.1007/s13659-025-00542-7 (PMC12405078; doi:10.1007/s13659-025-00542-7)
Supplement: Supplementary file 1 — Supplementary Material 1. [file 13659_2025_542_MOESM1_ESM.docx]

**Supplementary Materials**

A novel polysaccharide in *Polygonatum* *kingianum*: Structure elucidation, the activities of anti-inflammatory and the regulation of gut microbiota *in vitro*

Xiao Han^a^, Xin-Xiu Ren^a, b^, Dan-Yang Zhang^a^, Qin-Feng Guo^a^, Shi-Meng Li^a^, Zhi-Long Xiu^a^, Yue-Sheng Dong^a,^ *

a MOE Key Laboratory of Bio-Intelligent Manufacturing, School of Bioengineering, Dalian University of Technology, Dalian 116024, China.

b College of Basic Medical Sciences, Dalian Medical University, Dalian 116044, China.

* Corresponding author at School of Bioengineering, Dalian University of Technology, Dalian, Liaoning, China.

E-mail address: yshdong@dlut.edu.cn.

Telephone number: + 86-15909852985.

**Contents**


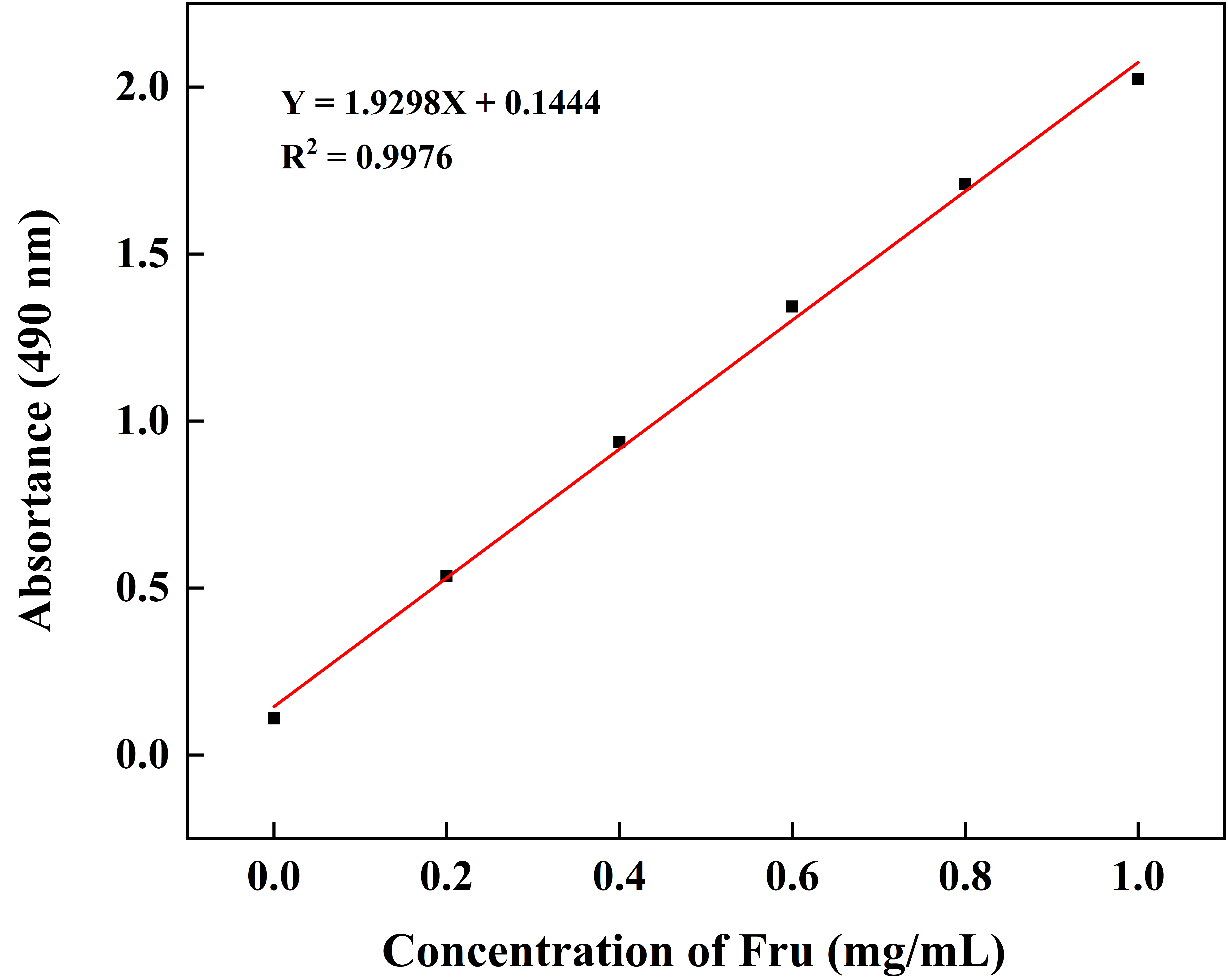


**Fig. S1** Standard curve for polysaccharide purity analysis (Fru as standard)


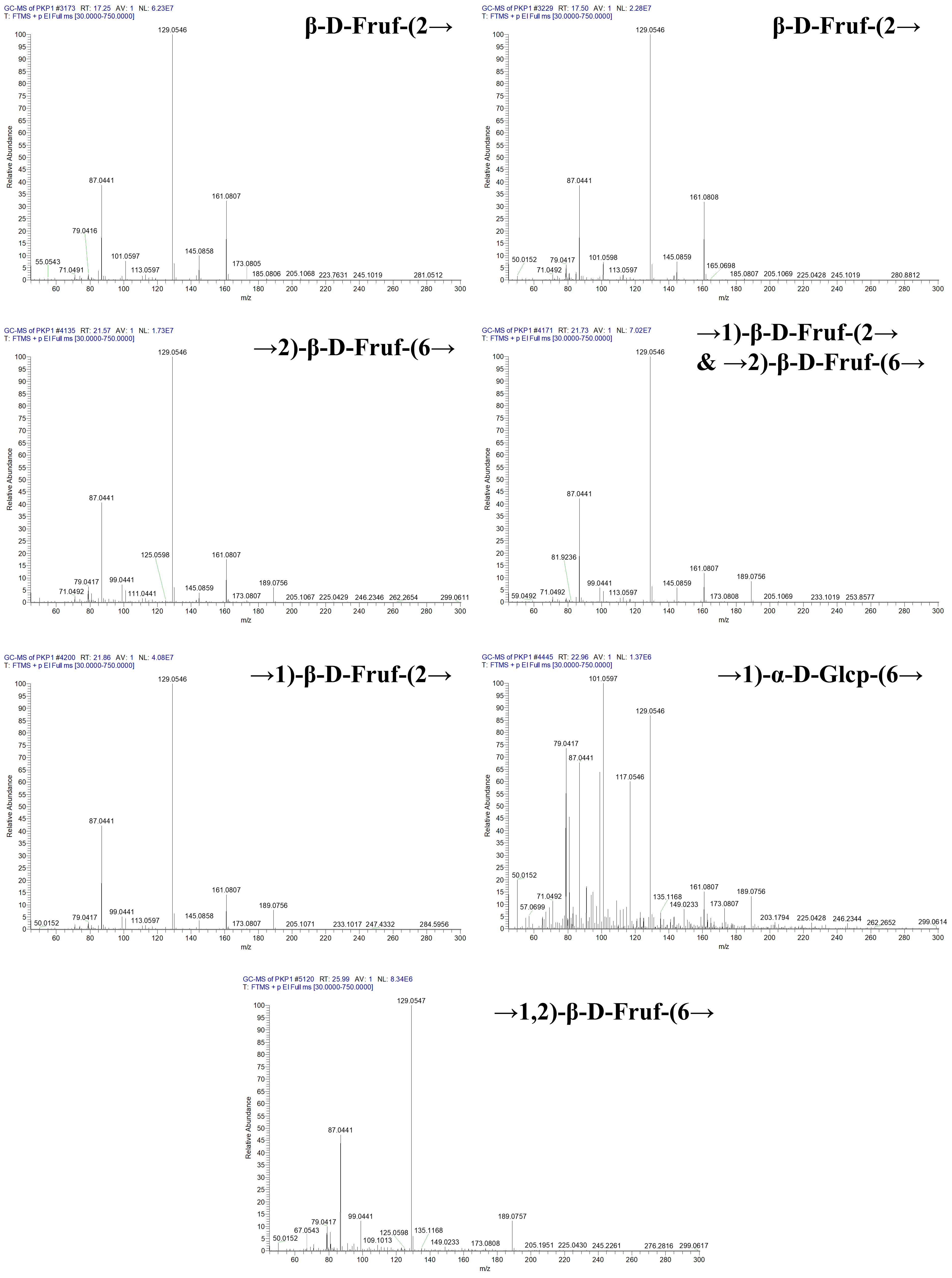


**Fig. S2** Characteristic ion fragments for PKP1 methylation analysis

**Table S1** Standard curve equations, linear range, and linear correlation coefficients for four types of SCFAs.

| Standard | Standard curve equations | linear ranges (mg/mL) | R^2^ |
| --- | --- | --- | --- |
| Acetic acid | y = 382898.79*x + 4422.37 | 0.1-3 | 0.9998 |
| Propionic acid | y = 719033.52*x + 1330.19 | 0.03-1 | 0.9999 |
| Isobutyric acid | y = 949911.08*x + 477.42 | 0.01-0.2 | 0.9994 |
| Butyric acid | y = 891129.90*x + 3559.55 | 0.01-0.4 | 0.9985 |
